# A village-matched evaluation of providing a local supplemental food during pregnancy in rural Bangladesh: a preliminary study

**DOI:** 10.1186/s12884-018-1915-x

**Published:** 2018-07-04

**Authors:** Briony Stevens, Kerrianne Watt, Julie Brimbecombe, Alan Clough, Jenni A. Judd, Daniel Lindsay

**Affiliations:** 1 College of Public Health, Medical and Veterinary Sciences, James Cook University, QLD, Townsville, Australia; 2 Department of Nutrition, Dietetics and Food, Faculty of Medicine, Nursing and Health Sciences, Monash University, Melbourne, Vic Australia; 3 Anton Breinl Centre for Health Systems Strengthening, James Cook University, QLD, Townsville, Australia; 4 Australian Institute of Tropical Health and Medicine, James Cook University, QLD, Townsville, Australia; 5 Centre for Research Excellence in the Prevention of Chronic Conditions in Rural and Remote Populations, James Cook University, QLD, Cairns, Australia; 6 College of Medicine and Dentistry, James Cook University, QLD, Townsville, Australia; 7 School of Health, Medical and Applied Sciences, Central Queensland University, QLD, Bundaberg, Australia

**Keywords:** Maternal undernutrition, Bangladesh, Low-income country, Balanced protein energy supplementation, Low birth weight

## Abstract

**Background:**

Prenatal balanced protein energy supplementation consumed by undernourished women improves mid-upper arm circumference in early infancy. This study aimed to identify whether locally produced maternal food-based supplementation improved anthropometric measures at birth and early infancy.

**Methods:**

A village-matched evaluation, applying principles of a cluster randomised controlled trial, of a locally produced supplemental food to 87 undernourished pregnant women. 12 villages (intervention: *n* = 8; control: *n* = 4) in Pirganj sub-district, Rangpur District, northern Bangladesh. Daily supplements were provided.

**Results:**

Anthropometric data at birth were available for 77 mother-infant dyads and longer-term infant growth data for 75 infants. Mid-upper arm circumference (MUAC) was significantly larger in infants of mothers in the intervention group compared with the control group at 6 months (*p* < 0.05). The mean birth weight in babies of supplemented mothers (mean: 2·91 kg; SD: 0·19) was higher than in babies of mothers in the control group (mean: 2·72 kg; SD: 0·13), and these changes persisted until 6 months. Also, the proportion of low birth weight babies in the intervention group was much lower (event rate = 0.04) than in the control group (event rate = 0.16). However, none of these differences were statistically significant (*p* > 0·05; most likely due to small sample size). The intervention reduced the risk of wasting at 6 months by 63.38% (RRR = 0.6338), and of low birth weight by 88·58% (RRR = 0.8858), with NNT of 2.22 and 6.32, respectively. Only three pregnant women require this intervention in order to prevent wasting at 6 months in one child, and seven need the intervention to prevent low birth weight of one child.

**Conclusions:**

Locally produced food-based balanced protein energy supplementation in undernourished pregnant women in northern Bangladesh resulted in larger MUAC in infants at 6 months. Further research, with larger sample sizes, is required to confirm the role of locally produced supplementation for undernourished pregnant women on weight and linear growth in newborns and infants.

**Trial registration:**

This research was registered with the ISRCTN registry (ISRCTN97447076). This project had human research ethical approval from the James Cook University (Australia) Ethics committee (H4498) and the Bangladesh Medical Research Council (BMRC/NREC/2010–2013/58).

**Electronic supplementary material:**

The online version of this article (10.1186/s12884-018-1915-x) contains supplementary material, which is available to authorized users.

## Background

Size at birth and growth in early infancy are important indicators of early childhood survival and health. Maternal undernutrition, an indicator of poor fetal growth, is associated with lowered birth weight [1]. Low birth weight babies have a substantially increased risk of stunting, infant mortality, and morbidity [2, 3]. Childhood undernutrition leads to increased susceptibility to infections and subsequent undernutrition [4]; shorter adult height [5]; decreased cognitive function [6]; and increased risk of maternal complications and chronic disease in later life [5, 7].

In a recent systematic review and meta-analysis, balanced protein energy supplementation among undernourished pregnant women was found to significantly improve birth weight in low and middle-income countries (*d* = 0·203, 95% CI, 0·03–0·38, *p* = 0·021) [8]. The impact on longer-term growth remains inconclusive because few randomised controlled trials (RCTs) have reported on this outcome [8]. An RCT in Indonesia showed a significant increase in height up to 60 months, and weight up to 24 months of age, with a greater effect at 9 and 12 months of age, respectively [9]. Positive findings have been reported in non-RCTs on longer-term growth from supplementation of undernourished pregnant women, especially when the supplement meets an energy gap [8, 10–12].

Although a number of studies have included food-based supplements using locally available and preferred foods [13–16], the evidence on effective approaches for the treatment of acute maternal malnutrition to improve growth outcomes at birth and early infancy is limited, and sustainability is rarely considered. The cost of food-based supplementation, even locally produced, is often beyond the reach of pregnant women in low-income countries, where undernutrition is most prevalent [17].

Bangladesh has among the highest rates of maternal and child undernutrition worldwide [18]. Maternal undernutrition affects one in three women (defined as a body mass index < 18·5 kg/m^2^) [19] and low birth weight (< 2·5 kg) affects one in five newborns [20]. Among children aged 6 to 59 months, stunting has declined from 51% in 2004 to 36% in 2014, and wasting has declined from 15 to 14% [21]. In rural Bangladesh, extreme poverty^1^ is three times higher than in urban areas, women are less likely to access antenatal and postnatal services, and children suffer from higher rates of chronic malnutrition (43 and 36% in rural and urban areas, respectively) [22, 23].

The purpose of this study was to identify whether a locally developed balanced protein energy supplementation for undernourished pregnant women in rural areas of northern Bangladesh, affected anthropometric measures at birth and during early infancy.

## Methods

### Setting and location

The study was conducted in the Pirganj sub-district of Rangpur District, northern Bangladesh. The Pirganj sub-district covers an area of 411·34 km^2^, consists of 332 villages, and has 385,499 inhabitants [24]. As in other areas of Bangladesh, Rangpur has a tropical monsoon climate with high temperatures, high humidity and heavy seasonal rainfall from June to November. The villages are rural with dirt roads and are often inaccessible during the wet season. Rangpur is reported to be more vulnerable to seasonal food insecurity than other areas of Bangladesh [25]. Rangpur’s main source of employment is agricultural labour though wages are low compared to neighbouring districts [26]. We previously identified that households were largely food insecure, that the dietary diversity was poor, and that households relied on homestead food production for both sustenance and income [27, 28].

### Study design

The study reported here was the third phase of a multiphase study design. All three phases were committed to local level applied research. The previous two phases were published accordingly [8, 27–30]. Phase one consisted of formative research exploring maternal dietary preferences, and barriers and enablers to healthy eating in a sub-sample of the study locations. Phase two involved the development of a locally produced prenatal food based supplement. To achieve this, a small business enterprise was established, packaging was designed, and a 30-day acceptability study conducted. As reported here, the third phase consists of a pilot study testing the effectiveness of the locally developed supplement.

A village-matched evaluation, using principles of a cluster randomised controlled trial, was conducted. Recruitment occurred from February 2013 to February 2015. Rangpur district was selected based on its rural location. Pirganj sub-district was purposively selected for this study from eight sub-districts in Rangpur. Two of 15 unions^2^ from Pirganj were selected: one randomly selected as the intervention site using computer-generated random numbers, and the other was matched to the intervention union to act as the control. From the intervention union, eight villages of a possible 24 were randomized to receive the intervention using computer-generated random numbers. From the control union, four villages of a possible 30 were purposively matched to the intervention villages. Criteria for matched controls included similarities between socio-economics, demographics and agricultural produce. The number of villages was determined based on population, estimated incidence of pregnant women across the study period, and the required sample size [19, 23, 24]. The estimated sample size was targeted to detect a reduction of the proportion of babies with LBW by 50% (from 38 to 19%), with 80% power and alpha = 0·05.

### Participants and recruitment

In the selected villages, the study recruited women of reproductive age who were (1) confirmed to be pregnant (by skilled health professional); (2) undernourished, as defined by a mid-upper-arm circumference (MUAC) ≤22·1 cm^3^^,^^4^; and (3) not in need of a medical referral. (4) Exclusion criteria included (1) the delivery of twins (only singleton deliveries included); (2) a delivery outside of the study period.

Information on the study recruitment and community nutrition volunteers has been published elsewhere [29]. In summary, trained community nutrition volunteers^5^ compiled lists of all pregnant women in the twelve villages and invited these women to participate. Eligible women were given a brief overview of the project and written or verbal (with thumbprint) consent to participate was obtained after the participants had heard the project information sheet read aloud in their local language. Verbal consent was also obtained from the leaders of each village to include their village in the study.

Eight female community nutrition volunteers and two (one male and one female) supervisors from the selected villages were trained on the basics of nutrition, and the study purpose and design. The community nutrition volunteers had at least a primary school education, spoke the local dialect, and were aged between 21 and 49 years.

Women in the intervention group received the intervention by visiting community nutrition volunteers at a designated community site on a daily basis. If the women were unable to visit the community nutrition volunteer, the volunteer would visit the household. All enrolled women in the intervention villages received one serving of the food-based supplement per day, within seven days of enrolment to term. The community nutrition volunteers closely monitored compliance to the food-based supplement at every visit through observation and discussion. Women from the control villages were unable to access the intervention supplement from the intervention villages due to the careful enrolment and follow-up process conducted by the community nutrition volunteers.

### Supplement

Details on how the supplement was developed are published elsewhere [30]. In summary, a small business enterprise comprising of local women was established, and a food-based balanced protein energy supplement was developed. The supplement consisted of 27% pigeon pea, 35% banana, 16% sugar, 9% peanuts, 6% whole milk powder, 6% sesame seeds and 1% iodised salt (Additional file 1: Table S1). The supplement was pretested, and the most acceptable version of the supplement was selected for use in this intervention study. The composition and energy content of the supplement was designed to meet the estimated energy gap of undernourished pregnant women, while the protein provided was less than 25% of the total energy content [31]. Using locally procured ingredients, the supplement was prepared at a single centre by village women.

### Nutrition screening, nutrition education, and antenatal and postnatal services

Women in intervention and control groups received identical services and support except for provision of the supplement. All pregnant women (regardless of study enrolment) in the intervention and control groups were screened for undernutrition through village-level monthly campaigns conducted by the community nutrition volunteers. Referral pathways with existing government- and NGO-supported ante-natal care (ANC) and post-natal care (PNC) services were established at the onset of the study.

Regardless of study group, or nutrition status, all pregnant women living in the villages selected to participate in this trial received iron-folic acid supplementation through access to routine ANC and PNC services. In addition, all women were tested for anaemia at a mobile clinic and were provided treatment if identified as anaemic. The partnership with the mobile clinic was established for the purpose of this study.

### Data collection

Upon enrolment, community nutrition volunteers collected the following data from participants: 1) background demographics; 2) household food security; 3) dietary diversity and 4) anthropometry. Household food insecurity was identified using the validated Food and Nutrition Technical Assistance (FANTA) Household Food Insecurity Access Scale (HFIAS) questionnaire (Version 3) [32, 33]. Women’s dietary diversity scores (WDDS) were determined using the validated Food and Agricultural Organisations (FAO) dietary diversity questionnaire [34, 35]. Dichotomous variables were created to indicate the presence (or absence) of each food group, then these were aggregated to compute a WDDS [35]. A higher HFIAS score indicates greater food insecurity, and lower WDDS indicates lower dietary diversity. The questionnaires were contextualised, translated into local terminology, back-translated and field tested prior to use.

The community nutrition volunteers measured height, weight and MUAC at participant enrolment and each month of the data collection period, using the standardised procedures recommended by the World Health Organisation [36, 37]. Height was measured to the nearest millimetre (mm) with community-made adult height boards, and weight to the nearest 100 g (g) with digital SECA scales. MUAC was measured to the nearest mm with adult MUAC tapes. Triplicate measurements were taken if a variation occurred between the two measurements. Maternal undernutrition was defined as MUAC ≤22·1 cm. The cut-off was determined after a review of the evidence and a discussion with organisations conducting nutrition programmes and research in Bangladesh [36].

At birth, skilled health professionals^6^ recorded newborn length to the nearest mm with a child length board (standardised UNICEF length/height board), and weight to the nearest 100 g with a beam-type scale (SECA baby-scales). Where a skilled health worker was not present or able to complete the measurement, community nutrition volunteers did so. Community nutrition volunteers recorded infant weight and MUAC at 1, 3, and 6 months of age, or until the end of the study. Weight at 6 months was measured using a calibrated digital scale (SECA). Birth measurements were taken at the place of birth within 24 h of delivery. Longer-term measurements were taken at a designated community space, or at the household. To ensure reliability, all anthropometric variables were measured twice by the community nutrition volunteers, and once by the clinic staff, if the birth occurred at a health centre. If the two measurements differed, a third measurement was obtained to verify the correct measure. All weighing scales were calibrated daily. Community nutrition volunteers recorded the occurrence of maternal mortality, miscarriages, stillbirths, and loss to follow-up. The procedure was the same for all participants, whether in the intervention or control group.

### Outcomes

To identify whether a locally produced food-based supplement improved birth and infant anthropometric measures, the primary outcomes included birth weight and longer-term infant growth (weight and MUAC) at 1, 3, and 6 months. The secondary outcomes were preterm birth (defined as birth at < 37 weeks of gestation), miscarriage (delivery of infant ≤20 weeks), stillbirth (delivery of an infant showing no signs of life ≥20 weeks), perinatal death (stillbirth or death of a live-born infant in the first 7 days after birth), and neonatal death (death within the first 28 days of life). We also measured acute malnutrition (wasting) in infants at 6 months. Wasting in infants was defined by a MUAC < 12·5 cm [38].

Compliance was calculated by dividing the total number of days the supplement was taken under direct observation, by the total number of days possible, i.e., the number of days between enrolment and delivery. We defined loss to follow-up as a participant leaving the study area for a period longer than 2 consecutive weeks or delivering their infant in a place outside the study area.

### Statistical methods

The Consolidated Standards of Reporting Trials (CONSORT) statement was used to ensure comprehensive reporting of this study. The data quality was ensured by quality checks associated with the data entry process, double entry, and data cleaning. Data management was conducted using Microsoft Excel. Statistical analyses were performed using the statistical software package IBM SPSS software, Version 23© (Armonk, NY, USA).

For categorical variables, between group differences were assessed as suggested by Donner and Klar (1994) [39]. Categories were treated as event rates, and these were calculated for each cluster (e.g., proportion of babies with low birth weight in each cluster). Independent samples t-tests were then used to compare intervention and control groups, taking into account the clustering. For numerical variables, means were calculated for each cluster, then independent samples t-tests were used to compare differences between intervention and control groups, accounting for clusters (as described in Campbell et al. 2000) [40]. A *p*-value of < 0·05 was considered statistically significant. To estimate the effect of the intervention, absolute measures of risk were also calculated. Specifically, Relative Risk Reduction (RRR), Absolute Risk Reduction (ARR), and Number Needed to Treat (NNT) were calculated in relation to the proportion of participants categorised as low birth weight (< 2·5 kg), and the proportion of participants with MUAC< 12·5 at 6 months.

### Ethics

This research was registered with the ISRCTN registry (ISRCTN97447076). This project had human research ethical approval from the James Cook University (Australia) Ethics committee (H4498) and the Bangladesh Medical Research Council (BMRC/NREC/2010–2013/58).

## Results

### Participation and recruitment

**Fig.1:**
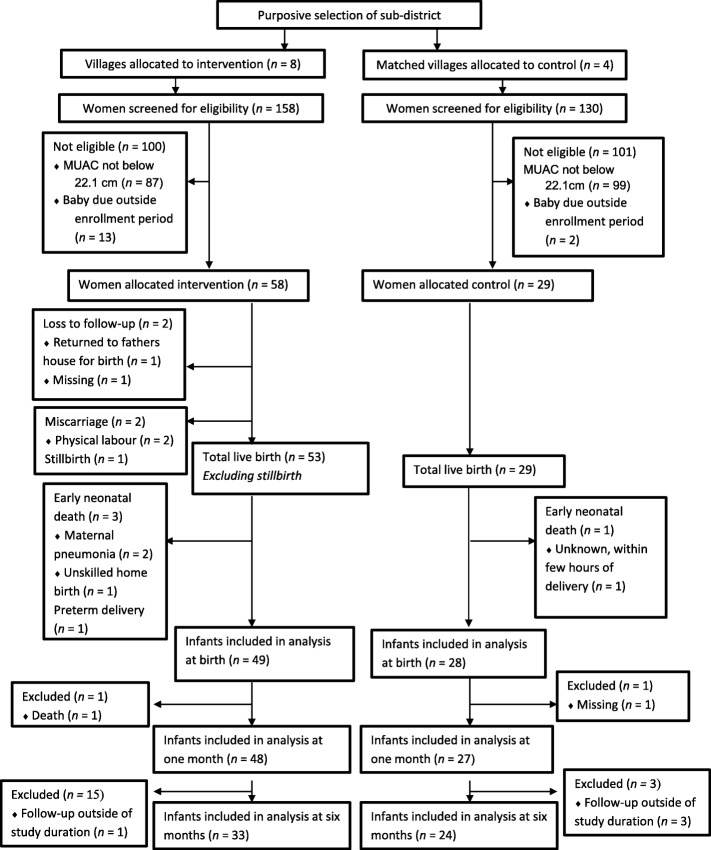
Profile of the study (modified from the CONSORT diagram)

The participation and recruitment data are presented in Fig. 1. From February 2013 to February 2015, 87 mothers aged 14 to 31 years were enrolled in the study: 58 in the intervention group and 29 in the control (Fig. 1). Birth weight outcome data were available for 77 of 87 (88·5%) pregnant women (intervention: *n* = 49, control: *n* = 28) and infant anthropometric outcome data were available for 75 children (intervention: *n* = 48, control: *n* = 27). All births were singleton. No caesarean sections were recorded for the mothers in either group. The main reason for missing infant anthropometric outcome data was that the duration of the data collection phase did not adequately allow for 6 months of follow up data to be collected from infants enrolled later in the study. Other reasons for missing longer-term growth data included mothers being unavailable to bring their child to the assessments due to household work or caring for their other children. The number of women lost to follow-up differed between groups (intervention: *n* = 2; control: *n* = 0). The reason that one woman was lost to follow-up was that she returned to her father’s village to give birth; the other was unknown. Due to low numbers of preterm birth, miscarriage, stillbirth, perinatal death, and neonatal death, analyses on secondary outcomes were not conducted. Instead, these data were excluded from analyses (intervention: *n* = 7; control: *n* = 1). Two cases were excluded from longer-term analyses; one due to death (intervention), and one missing for unknown reasons (control). All infants identified with moderate or severe acute malnutrition were immediately referred to community health centres for treatment^7^.

### Baseline characteristics and compliance

The background characteristics of the 87 enrolled women are presented in Table 1. There were no differences on demographic or anthropometric characteristics, dietary or food security, between the intervention and control groups at baseline (*p* > 0·05), except that women in the intervention group were more religiously diverse (Muslim, Hindu, and Christian compared with 100% Muslim in the controls; *p* < 0·01). The mean age of the women was 22·6 years (SD: 5·3). Most women attended school, with the majority reaching either a primary or lower secondary education level (37·6% and 47·1%, respectively). The mean height of the women was 148·5 cm (SD: 5·5), with more than half below 148 cm (52·6%), indicating chronic malnutrition. Most pregnancies were identified in the first or early second trimester (29·9% and 41·4%, respectively).

**Table 1:** Background characteristics of participants according to study group (*N =* 87)^a^

	Intervention group (*n* = 58)				Control group (*n* = 29)			
Background characteristics	n	%	Median	IQR	n	%	Median	IQR
Maternal age (years)	31		21	10	18		22·5	7
Trimester								
First	23	41·1			10	38·5		
Second	26	46·4			13	50·0		
Third	7	12·5			3	11·5		
Average household (HH) size	58		3	2	29		3	2
Food purchase decision-maker								
Self	17	29·3			9	31·0		
Husband	33	56·9			17	58·6		
In-laws	8	13·8			3	10·3		
Own land for cultivation	30	51·7			19	67·9		
School attendance								
Never attended	3	5·3			2	7·1		
Primary	20	35·1			12	42·9		
Secondary	34	59·6			14	50·0		
Religion**								
Islam	39	67·2			29	100		
Hindu	13	22·4						
Christianity	6	10·3						
Parity								
0	25	43·1			16	55·2		
1	26	44·8			12	41·4		
2	5	8·6			0	0		
≥ 3	2	3·4			1	3·4		
Stillbirth	0	0			1	3·4		
Miscarriage	5	8·6			1	3·4		
Anthropometrics								
Maternal height (cm)	58		148	7.5	28		147	10·5
Maternal height < 148 cm	31	53·4			16	55·2		
Dietary diversity score	58		5	1	29		5	1·5
Food security status								
Food secure	1	2·1			3	10·7		
Food insecure	46	97·9			25	89·3		

^a^Trimester of pregnancy was determined based on last menstrual period (LMP)

** = *p* < 0.05

Compliance was high among the intervention group; almost all women consumed the full supplement on a daily basis (*n* = 57). One woman did not consume the supplement for a one-month duration during the third trimester, as she was not reachable and therefore did not receive the supplement. No women refused the supplement.

### Main results

#### Birth weight

The primary outcome data are presented in Table 2. Birth weight was higher in the intervention group (mean: 2·91; SD: 0·19) compared to the control group (mean: 2·72; SD: 0·13) but this was not significant (*p* > 0·05). We grouped birth weight into two categories: healthy birth weight (≥2·50 kg) and low birth weight (< 2·50 kg). Low birth weight occurred much less frequently in the intervention group (event rate = 0.04; ±0.10) compared to the control group (event rate = 0·16 ± 0·18), but this was not significant (*p* > 0·05).

**Table 2:** Weight (birth, 1, 3, and 6 months) and MUAC (6 months) of Intervention and Control

	Intervention group (*n* = 8)		Control group (*n* = 4)			
Outcome	Mean	SD	Mean	SD	P	
Weight (kg)						
Birth weight	2·91	0·19	2·72	0·13	0·13	
1 month	3·88	0·35	3·44	0·33	0·06	
3 month	5·64	0·74	4·87	0·49	0·09	
6 month	7·01	0·89	6·53	0·67	0·37	
MUAC (cm)						
6 month	12·83	0·62	12·01	0·21	0·03	
Low birth weight^a^	0·04	0.10	0·16	0.18	0·28	
MUAC< 12.5^a^	0·26	0·22	0·71	0·22	0·01	

^a^Event rate

Note: Village means for birth weight, weight at 1, 3 and 6 months, and MUAC at 6 months are shown in Additional file 2: Table S2

#### Infant weight

Although mean weight in the intervention group was higher than the control group at 1, 3, and 6 months, these differences were not statistically significant (*p* > 0·05).

#### MUAC

The mean MUAC was significantly larger in the intervention group compared to the control group at 6 months (intervention mean: 12·83, SD: 0·62; control mean: 12·01, SD: 0·21 at 6 months; *p* < 0·05).

We conducted analyses to explore the event rate of wasting at 6 months in the intervention and control groups. The event rate was significantly lower in the intervention group compared to the control group (intervention mean: 0·26, SD: 0·22; control mean 0·71, SD 0·21; *p* < 0·05).

Absolute measures of risk were calculated to further describe the effect of the intervention, based on the proportion of participants categorized as low birth weight, and the proportion with MUAC< 12·5 at 6 months. The intervention reduced the risk of low birth weight by 88·58% (RRR = 0·8858), and MUAC < 12·5 at 6 months by 63·38% (RRR = 0·6338). The absolute risk reduction was 15·82% for LBW, and 45% for MUAC< 12·5. The Number Needed to Treat (NNT) was 6·32 (low birthweight) and 2.22 (MUAC< 12·5). That is, 7 pregnant women need to experience this intervention in order to prevent low birth weight in one child, and 3 pregnant women need to experience the intervention in order to prevent wasting at 6 months in one child.

## Discussion

In northern Bangladesh, we found that daily supplementation with a locally produced food-based balanced protein energy supplement during pregnancy among undernourished women resulted in infants with significantly larger MUAC measurements at 6 months and subsequently a lower event rate of wasting.

Although a clear trend was observed of heavier babies at birth to 6 months in the intervention group compared to the control group, the difference was not significant (*p* > 0·05).

MUAC reflects protein reserves and thus lean mass [41]. MUAC is an internationally recognised independent diagnostic criteria for acute malnutrition and is commonly used in the identification of children with a high risk of death who are in need of treatment [42]. Among pregnant women, MUAC cut-offs of < 21 and < 23 cm indicate risk of low birth weight [36]. Our data demonstrated that locally produced food-based balanced protein energy supplementation during pregnancy may contribute to preventing acute malnutrition in infants at 6 months of age as MUAC measurements were larger in the intervention group compared to the control group. This finding is supported by several other studies showing an association between birth weight and later lean mass in children [43, 44]. A subsequent novel finding of our research was the noticeable difference in the event rate of wasting in the intervention group compared to the control group. These findings align with those reported by Mora and colleagues, who identified a lower proportion of children with severe acute malnutrition in the intervention group compared to their control group [45, 46]. Our data suggest that the fetal growth period is an important determinant of infant body composition. Importantly, only 3 pregnant women need to experience the intervention in order to prevent wasting at 6 months in one child.

While there was a trend in favour of the intervention group, our study found that locally produced balanced protein energy supplementation did not significantly reduce the proportion of low birth weight babies among undernourished pregnant women. The most likely reason for not seeing a significant difference between the two groups is due to the small sample size. These data show that the intervention reduced the risk of low birth weight by 88·58% (RRR = 0·8858), and that only seven pregnant women require this intervention in order to prevent low birth weight of one child. This finding is supported by a recent systematic literature review on balanced protein supplementation among undernourished pregnant women in low- and middle- income countries [8]. A recent study in Malawi among moderately malnourished pregnant women reported that newborn birth weight was similar across intervention groups that received Ready to Use Supplementary Food (RUSF), Corn Soya Blend Plus (CSB+) with UNICEF/WHO/UNU International Multiple Micronutrient Preparation (UNIMMAP), and CSB+ with IFA, but the incidence of newborns with a birthweight < 2.4 kg was higher in the CSB+ with UNIMMAP group than the other groups [47]. In addition, a study in Burkina Faso reported no effects on birth weight and suggested that this may have been due to the provision of multiple micronutrient supplementation to the intervention and control groups (which is also known to increase birth weight), the targeting of all pregnant women (nourished and undernourished), or due to the energy content of the supplement [12]. Our study provided iron-folic acid, nutrition education and enabled access to ANC and PNC services to both groups. In our study, we enrolled undernourished women only and provided a high-energy supplement. It has recently been suggested that multiple micronutrient supplements have more of an effect on birth weight than iron-folic alone [48]. We did not identify an association between maternal supplementation and longer-term infant weight gain. However, we identified a trend for heavier infants in the intervention group from birth until 6 months. The most likely reason for not seeing a significant difference between the two groups may have been the study size and design, which resulted in our sample size decreasing with time.

Finally, our study highlighted that locally produced food-based balanced protein energy supplementation for undernourished pregnant women can be produced at a local level. While our finding is limited to the context of northern Bangladesh, similar studies have demonstrated that RUSF and Ready to Use Therapeutic Food (RUTF) can be locally produced and sustainable [49, 50].

Our study had a number of limitations. First, we calculated our sample size based on the prevalence and estimated incidence using the most recent Demographic Health Survey data [19]. Our calculation overestimated the number of women who would be enrolled in this study. In addition, the study design included the enrolment of pregnant women with subsequent follow-up over a 24-month period only. Funding availability largely influenced this. These factors resulted in a gradual decrease in the sample size, as we were unable to follow every infant born to an enrolled woman until 6 months of age. Further, the number of participants in each cluster, and in the overall study, was relatively low. This low sample size is a likely explanation for the lack of observed statistical significance in some of the results (e.g., birthweight), due to type 2 error. Second, it was initially planned that infant linear growth would be recorded. However, due to delays in the procurement of infant height/length boards, we were unable to include these data in the study. Third, due to the sensitivity around death, the volunteers did not record anthropometric data for perinatal deaths. Fourth, the monthly nutrition screening included a question on whether the mother experienced an illness over the last 14 days, which may have resulted in recall bias. An illness has the potential to affect anthropometric measurements, and may require referral to certain health services. Mothers may have forgotten an illness or when it occurred. Fifth, self-report may have been an issue for some of the measures, such as questions on household food security and dietary diversity. The monthly screening questions on whether the mother had received any other form of assistance from an NGO or other organisation, etc. In population groups where food assistance or developmental aid assistance are frequent, participants may over-report food insecurity and under-report dietary diversity with the expectation of receiving assistance. Conversely, participants may modify their responses based on social desirability. Sixth, we did not record whether participants were affected by chronic diseases that are known risk factors for Intrauterine Growth Restriction (IUGR) and LBW. Therefore, we cannot rule out that possible alternative explanations to our findings exist. However, such variables were considered when matching the control villages to the intervention villages. Lastly, despite the active home visits by the community nutrition volunteers, the proportion of women included early in pregnancy was lower than desired, a limitation that has been experienced by others [12].

## Conclusion

In Rangpur district in northern Bangladesh, locally produced balanced protein energy supplementation, using a community-based small business enterprise, reduced wasting in children at 6 months when targeted to undernourished pregnant women. Further, this research illustrates how communities can be empowered to identify and address maternal undernutrition. The intervention reduced the risk of wasting at 6 months by 63.38%, and of low birth weight by 88·58%. Only three pregnant women require this intervention in order to prevent wasting at 6 months in one child, and seven need the intervention to prevent low birth weight of one child. This supplementation thus contributed to better development outcomes for the mother and child. Our study findings highlight that programme decision-makers have alternatives to the commonly used Ready to Use Foods (RUFs) to treat acute maternal malnutrition, which may not be sustainable, cost effective or acceptable to some populations. While our study identified that the supplementation improved infant lean mass, we recommend that studies with larger sample sizes further explore this association. This study can inform future studies, with larger sample sizes, that aim to investigate the effect of maternal supplementation on longer-term infant growth (weight, length and MUAC).

### Key messages

- Effective balanced protein energy supplementation for undernourished pregnant women can be locally produced.
- Communities can be empowered to develop effective balanced protein energy supplementation, identify maternal acute malnutrition, and provide treatment.
- Daily supplementation with a locally produced food-based balanced protein energy supplementation during pregnancy may contribute to reducing acute malnutrition in infants at 6 months of age. Further studies with larger sample sizes are required to explore this association.
- Further research using larger sample sizes is required to explore the effect of daily supplementation using locally produced food-based balanced protein energy supplementation during pregnancy among undernourished women on a baby’s weight at birth ad early infancy.
- Findings from this study can inform, guide and motivate policies by providing evidence on a sustainable nutrition intervention that improves birth weight.

## Additional files

Additional file 1: **Table S1.** Composition of the food-based balanced protein energy supplement per serving. (DOCX 8 kb)

Additional file 2: **Table S2.** Village means for birth weight, weight at 1, 3, and 6, months, and MUAC at 6 months. (DOCX 9 kb)

## Abbreviations

ARR: Absolute risk reduction
BMRC: Bangladesh medical research council
CI: Confidence interval
CONSORT: Consolidated standard reporting of trials
FANTA: Food and nutrition technical assistance
FAO: Food and agricultural organisation
G: Grams
HFIAS: Household food insecurity access scale
IQR: Interquartile range
ISRCTN: International standard registered clinical social study number
MM: millimetres
MUAC: Mid-upper arm circumference
NNT: Number needed to treat
*P*-value: Probability
RCT: Randomised controlled trial
RRR: Relative risk reduction
SD: Standard deviation
WDDS: Womens dietary diversity scores

## Footnotes

1 Defined as earning less than $1.25 per day by the World Bank. See Ravallion, Chen, and Sangraula (2009) for further information on how the $1.25-a-day international poverty line was derived [22].

2 Union is defined as the smallest rural administrative and local government unit in Bangladesh.

3 MUAC was the preferred indicator to identify maternal undernutrition based on its association with low birth weight. Ververs MT, Antierens A, Sackl A, Staderini N, Captier V. Which anthropometric indicators identify a pregnant woman as acutely malnourished and predict adverse birth outcomes in the humanitarian context? PLoS currents. 2013;5; WHO. Maternal anthropometry and pregnancy outcomes. A WHO Collaborative Study. Bulletin of the World Health Organization. 1995.

4 Maternal undernutrition was defined by a MUAC ≤22·1 cm. The MUAC cut-off selected aligns with that commonly used by NGOs in Bangladesh at the time this study was designed.

5 Volunteers received a monthly stipend to compensate for time spent on this project and attended multiple trainings associated with this study.

6 Defined as those that have received formal training. In addition, skilled health professionals received additional anthropometric training for the purpose of this study.

7 World Vision (NGO) supported malnutrition services at the health centres located in the villages that participated in this study.

## References

[1] Stein AD, Zybert PA, van de Bor M, Lumey LH (2004). Intrauterine famine exposure and body proportions at birth: the Dutch hunger winter. Int J Epidemiol.

[2] Martorell R, Ramakrishnan U, Schroeder DG, Melgar P, Neufeld L (1998). Intrauterine growth retardation, body size, body composition and physical performance in adolescence. Eur J Clin Nutr.

[3] Katz J, Lee AC, Kozuki N, Lawn JE, Cousens S, Blencowe H, Ezzati M, Bhutta ZA, Marchant T, Willey BA (2013). Mortality risk in preterm and small- for-gestational-age infants in low-income and middle-income countries: a pooled country analysis. Lancet.

[4] Rytter MJ, Kolte L, Briend A, Friis H, Christensen VB (2014). The immune system in children with malnutrition--a systematic review. PLoS One.

[5] Victora CG, Adair L, Fall C, Hallal PC, Martorell R, Richter L, Sachdev HS, Maternal, Child Undernutrition Study G (2008). Maternal and child undernutrition: consequences for adult health and human capital. Lancet.

[6] Pitcher JB, Henderson-Smart DJ, Robinson JS (2006). Prenatal programming of human motor function. Volume 573.

[7] Harder T, Rodekamp E, Schellong K, Dudenhausen JW, Plagemann A (2007). Birth weight and subsequent risk of type 2 diabetes: a meta-analysis. Am J Epidemiol.

[8] Stevens B, Buettner P, Watt K, Clough A, Brimblecombe J, Judd J. The effect of balanced protein energy supplementation in undernourished pregnant women and child physical growth in low- and middle-income countries: a systematic review and meta- analysis. Matern Child Nutr. 2015; 10.1111/mcn.12183 PMC6860195 25857334

[9] Kusin JA, Kardjati S, Houtkooper JM, Renqvist UH (1992). Energy supplementation during pregnancy and postnatal growth. Lancet.

[10] Winkvist A, Habicht JP, Rasmussen KM (1998). **Linking maternal and infant benefits of a nutritional supplement during pregnancy and lactation**. Am J Clin Nutr.

[11] Tofail F, Persson LA, El Arifeen S, Hamadani JD, Mehrin F, Ridout D, Ekstrom EC, Huda SN, Grantham-McGregor SM (2008). Effects of prenatal food and micronutrient supplementation on infant development: a randomized trial from the maternal and infant nutrition interventions, Matlab (MINIMat) study. Am J Clin Nutr.

[12] Huybregts L, Roberfroid D, Lanou H, Menten J, Meda N, Van Camp J, Kolsteren P (2009). Prenatal food supplementation fortified with multiple micronutrients increases birth length: a randomized controlled trial in rural Burkina Faso. Am J Clin Nutr.

[13] Adu-Afarwuah S, Lartey A, Zeilani M, Dewey KG (2011). Acceptability of lipid- based nutrient supplements (LNS) among Ghanaian infants and pregnant or lactating women. Matern Child Nutr.

[14] Young SL, Blanco I, Hernandez-Cordero S, Pelto GH, Neufeld LM (2010). Organoleptic properties, ease of use, and perceived health effects are determinants of acceptability of micronutrient supplements among poor Mexican women. J Nutr.

[15] Ahmed T, Choudhury N, Hossain MI, Tangsuphoom N, Islam MM, de Pee S, Steiger G, Fuli R, Sarker SA, Parveen M (2014). Development and acceptability testing of ready-to-use supplementary food made from locally available food ingredients in Bangladesh. BMC Pediatr.

[16] Phuka J, Ashorn U, Ashorn P, Zeilani M, Cheung YB, Dewey KG, Manary M, Maleta K (2011). Acceptability of three novel lipid-based nutrient supplements among Malawian infants and their caregivers. Matern Child Nutr.

[17] Manary M. Local production and provision of ready-to-use therapeutic food for the treatment of severe childhood malnutrition. WHO technical background papers. 2006; 10.1177/15648265060273S305 17076214

[18] UNICEF (2009). The state of the world's children : special edition.

[19] NIPORT (2009). Bangladesh demographic and health survey.

[20] Ahmed T, Mahfuz M, Ireen S, Ahmed AM, Rahman S, Islam MM, Alam N, Hossain MI, Rahman SM, Ali MM (2012). Nutrition of children and women in Bangladesh: trends and directions for the future. J Health Popul Nutr.

[21] NIPORT, Associates ma, international I (2015). Bangladesh demographic and health survey 2014: key indicators.

[22] Ravallion M, Chen S, Sangraula P. Dollar a day revisited. The World Bank Economic Review. 2009;

[23] NIPORT (2012). Bangladesh demographic and health survey 2011: preliminary report.

[24] BBS: Bangladesh - Population and Housing Census. In. Bangladesh: Bangladesh Bureau of Statistics - Statistics and Informatics Division, Ministry of Planning; 2011.

[25] WFP: Bangladesh food security brief. In. Dhaka, Bangadesh; 2005.

[26] Zug S: Monga-seasonal food insecurity in Bangladesh -bringing the information together. The Journal of Social Studies, Centre for Social Studies (CSS), Dhaka 2006, No.111(Jul-Sep 2006).

[27] Stevens B, Clough A, Brimbecombe J, Watt K, Judd J. The use of a modified version of Photovoice to identify maternal dietary consumption enablers and barriers in northern Bangladesh. Int J Food, Nutrition and Public Health. 2016;8(1)

[28] Stevens B, Watt K, Clough A, Judd J, Brimblecombe J. An exploration of maternal dietary diversity and household food security in undernourished pregnant women living in northern Bangladesh. Int J Food, Nutrition and Public Health. 2015;7(2)

[29] Stevens B, Watt K, Brimbecombe J, Clough A, Judd J, Lindsay D. The role of seasonality on the diet and household food security of pregnant women living in rural Bangladesh: a cross-sectional study. Public Health Nutr. 2016:1–9. 10.1017/S136898001600183X PMC10261298 27573667

[30] Stevens B, Watt K, Brimblecombe J, Clough A, Judd J. Development of a locally produced balanced protein energy food-based supplement, and its acceptance by undernourished pregnant women in northern Bangladesh. Journal of Hunger & Environmental Nutrition.

[31] Ota E, Tobe-Gai R, Mori R, Farrar D (2012). Antenatal dietary advice and supplementation to increase energy and protein intake. Cochrane database of systematic reviews (Online).

[32] Coates J, Swindale A, Bilinsky P (2007). Household food insecurity access scale (HFIAS) for measurement of household food access: Indicator guide (v. 3). Washington, D.C.: food and nutrition technical assistance project, academy for educational Development.

[33] Jones AD, Ngure FM, Pelto G, Young SL (2013). What are we assessing when we measure food security? A compendium and review of current metrics. Adv Nutr (Bethesda).

[34] Arimond M, Wiesmann D, Becquey E, Carriquiry A, Daniels MC, Deitchler M, Fanou-Fogny N, Joseph ML, Kennedy G, Martin-Prevel Y (2010). Simple food group diversity indicators predict micronutrient adequacy of Women's diets in 5 diverse, resource-poor settings. J Nutr.

[35] Kennedy G, Ballard T, Dop M. Guidelines for measuring household and individual dietary diversity. In Rome: FAO. 2011;

[36] WHO: Maternal anthropometry and pregnancy outcomes. A WHO collaborative study**.** *Bulletin of the World Health Organization* 1995, 73 Suppl:1–98. PMC2486648 8529277

[37] WHO (1995). Physical status: the use and interpretation of anthropometry. Report of a WHO Expert Committee WHO Technical Report Series *No 854*.

[38] UNHCR/WFP (2011). Guidelines for Selective Feeding: the Management of Malnutrition in Emergencies.

[39] Donner A, Klar N (1994). Methods for comparing event rates in intervention studies when the unit of allocation is a cluster. American Journal of Epi.

[40] Campbell M, Mollison J, Steen N, Grimshaw J, Eccles M (2000). Analysis of cluster randomized controlled trials in primary care: a practical approach. Fam Pract.

[41] Ververs MT, Antierens A, Sackl A, Staderini N, Captier V. Which anthropometric indicators identify a pregnant woman as acutely malnourished and predict adverse birth outcomes in the humanitarian context? PLoS currents. 2013;5 10.1371/currents.dis.54a8b618c1bc031ea140e3f2934599c8 PMC3682760 23787989

[42] Briend A, Maire B, Fontaine O, Garenne M (2012). Mid-upper arm circumference and weight-for-height to identify high-risk malnourished under-five children. Matern Child Nutr.

[43] Wells JC, Hallal PC, Wright A, Singhal A, Victora CG (2005). Fetal, infant and childhood growth: relationships with body composition in Brazilian boys aged 9 years. Int J Obes.

[44] Hediger ML, Overpeck MD, Kuczmarski RJ, McGlynn A, Maurer KR, Davis WW (1998). Muscularity and fatness of infants and young children born small- or large-for-gestational-age. Pediatrics.

[45] Mora JO, Herrera MG, Suescun J, de Navarro L, Wagner M (1981). The effects of nutritional supplementation on physical growth of children at risk of malnutrition. Am J Clin Nutr.

[46] Mora JO, Paredes B, Wagner M, Navarro L, Suescun J, Christiansen N, Herrera MG (1979). Nutritional supplementation and the outcome of pregnancy. I. Birth weight. Am J Clin Nutr.

[47] Callaghan-Gillespie M, Schaffner AA, Garcia P, Fry J, Eckert R, Malek S, Trehan I, Thakwalakwa C, Maleta KM, Manary MJ (2017). Trial of ready-to-use supplemental food and corn-soy blend in pregnant Malawian women with moderate malnutrition: a randomized controlled clinical trial. Am J Clin Nutr.

[48] Haider BA, Bhutta ZA (2015). Multiple-micronutrient supplementation for women during pregnancy. Cochrane database of systematic reviews (Online).

[49] Weber JM, Ryan KN, Tandon R, Mathur M, Girma T, Steiner-Asiedu M, Saalia F, Zaidi S, et al. Acceptability of locally produced ready-to-use therapeutic foods in Ethiopia, Ghana, Pakistan and India. Matern Child Nutr. 2017: Apr;13(2) 10.1111/mcn.12250 PMC6865965 26776270

[50] Lagrone L, Cole S, Schondelmeyer A, Maleta K, Manary MJ (2010). Locally produced ready-to-use supplementary food is an effective treatment of moderate acute malnutrition in an operational setting. Ann Trop Paediatr.

