# Supplementary material for: A village-matched evaluation of providing a local supplemental food during pregnancy in rural Bangladesh: a preliminary study
Source: BMC Pregnancy Childbirth. 2018 Jul 4;18:286. doi: 10.1186/s12884-018-1915-x (PMC6030796; doi:10.1186/s12884-018-1915-x)
Supplement: Supplementary file 1 — Table S1. Composition of the food-based balanced protein energy supplement per serving. (DOCX 8 kb) [file 12884_2018_1915_MOESM1_ESM.docx]

Additional file 1

Supplementary Table 1. Composition of the food-based balanced protein energy supplement per serving

| Serving size 173 g Servings per packet 1 | | |  |
| --- | --- | --- | --- |
| Ingredients: Pigeon pea (48 g), banana (60 g), white sugar (28 g), peanuts (15 g), whole milk powder (10 g), sesame seeds (10 g), iodised salt (2 g) | | |  |
| Nutrient | Amount per serving | Percentage of daily value (%) | |
| Calories (Kcal) | 522 | 22 | |
| Protein (g) | 19.5 | 31 | |
| Fat (g) | 15.8 | 30 | |
| Calcium (mg) | 270 | 45 | |
| Iron mg) | 5.5 | 6 | |
| Iodine (µg) | 128 | 64 | |
| Vitamin A (µg RE) | 105 | 18 | |
| Thiamine (mg) | 0.53 | 53 | |
| Riboflavin (mg) | 0.27 | 18 | |
| Niacin (mg) | 6.1 | 38 | |
| Vitamin C (mg) | 5 | 11 | |
