# Supplementary material for: A village-matched evaluation of providing a local supplemental food during pregnancy in rural Bangladesh: a preliminary study
Source: BMC Pregnancy Childbirth. 2018 Jul 4;18:286. doi: 10.1186/s12884-018-1915-x (PMC6030796; doi:10.1186/s12884-018-1915-x)
Supplement: Supplementary file 2 — Table S2. Village means for birth weight, weight at 1, 3, and 6, months, and MUAC at 6 months. (DOCX 9 kb) [file 12884_2018_1915_MOESM2_ESM.docx]

**Additional file 2**

**Supplementary Table 2.** Village means for birth weight, weight at 1, 3, and 6, months, and MUAC at 6 months

|  | **Intervention**  (village) | | | | | | | | **Control**  (village) | | | |
| --- | --- | --- | --- | --- | --- | --- | --- | --- | --- | --- | --- | --- |
| **Outcome** | 1 | 2 | 3 | 4 | 5 | 6 | 7 | 8 | 1 | 2 | 3 | 4 |
| **Weight (kg)** |  |  |  |  |  |  |  |  |  |  |  |  |
| Birth weight | 3·20 | 2·78 | 2·93 | 2·61 | 3·00 | 3·10 | 2·70 | 2·94 | 2·75 | 2·80 | 2·54 | 2·84 |
| 1 month | 4·44 | 3·50 | 3·88 | 3·59 | 4·13 | 4·13 | 3·47 | 3·96 | 3·47 | 3·00 | 3·52 | 3·79 |
| 3 month | 6·05 | 4·81 | 5·15 | 5·43 | 6·55 | 6·80 | 4·94 | 5·38 | 4·38 | 4·60 | 5·01 | 5·50 |
| 6 month | 7·63 | 6·29 | 5·90 | 6·55 | 7·85 | - | 6·63 | 8·25 | 6·92 | 5·60 | 6·50 | 7·09 |
| **MUAC (cm)** |  |  |  |  |  |  |  |  |  |  |  |  |
| 6 months | 12·44 | 13·79 | 12·50 | 12·77 | 12·10 | - | 13·57 | 12·65 | 11·80 | 12·00 | 11·94 | 12·30 |
